# Key attributes of successful research institutes

**DOI:** 10.1371/journal.pbio.3002267

**Published:** 2023-09-05

**Authors:** Frank Bradke, Aidan Maartens, Sarah A. Teichmann

**Affiliations:** 1 Laboratory for Axon Growth and Regeneration, German Center for Neurodegenerative Diseases (DZNE), Bonn, Germany; 2 Wellcome Sanger Institute, Hinxton, Cambridge, United Kingdom; 3 Cavendish Laboratory, University of Cambridge, Cambridge, United Kingdom

## Abstract

Science does not take place in a vacuum: The physical and social workplace has a profound influence on scientific discoveries. Everyone at a research institute can contribute to its scientific output and productivity, from faculty research groups to facilities and platforms staff to administration and corporate services. Although the researchers addressing exciting scientific questions are key, their efforts can be fostered and directed by the overarching strategy of the institute, interconnection with facilities and platforms, and strong and directed support of the administration and corporate services. Everybody counts and everybody should be empowered to contribute. But what are the characteristics that make scientific organizations and their people flourish? This Essay looks at the structure and culture of successful research institutes, laying out different operational strategies and highlighting points that need be taken into consideration during their implementation.

## Abstract 2

What are the features that make a research institute and its people flourish? This Essay explores the organization and culture of successful research institutes and looks at the lessons that can be learned from them.

## Introduction

When scientists start working at a research institute, they quickly realize that they are not just inside the bubble of their own laboratory, but are part of a bigger ecosystem. In the words of John Donne, “no man is an island,” and this rings true of scientists and research groups as well. The culture of a research institute, its scientific standards, its social cohesion, and its funding framework are critical to its research output. But what are the key ingredients for a thriving institute?

Research institutes across the globe have put considerable efforts into building environments that facilitate the conception and exploitation of novel scientific ideas. A critical aspect of these environments is educational: throughout their careers, scientists continuously learn from each other by emulation, discussion, collaboration, and competition. This reflects the proverb that states “it takes a village to raise a child.” A research institute provides exactly this. It is the whole village—with all its constituent residents—in which scientists develop, formulate, and pursue their ideas, but also from which they emerge to join other scientific communities worldwide.

In this Essay, we explore strategies that have helped institutes develop into particularly successful and special places. This analysis is based on our almost 4 decades of experience working as group leaders and program heads at research institutes and in sitting on institutional scientific advisory boards and external review panels. These experiences spurred hours of discussion among ourselves and with colleagues, mentors, and mentees about the underlying philosophy, research culture, and organizational structures of successful, adaptable, and forward-looking research institutes. Here, we share our perspectives from these informal discussions. There is an inevitable geographical bias towards those institutions that we have interacted with in one way or another, but we hope the lessons we have gleaned have relevance to research institutes across the globe.

## Research institute organization and culture

The philosophy behind most research institutes is to free up scientists’ time to focus on research, with little or no teaching and the provision of internal funding. Many institutes do run world-class postgraduate training programs in affiliation with universities, but the focus tends to be on training through research projects rather than theoretical lectures. Research institutes often encourage interdisciplinarity and collaboration, and many form a structure to promote the intersection of disciplines such as biology with technologies and methods rooted in physics, chemistry, or computer science, for example. Research institutes have been remarkably successful, as demonstrated by their major contributions to ground-breaking discoveries such as the classification of developmental patterning genes [1], the determination of high-resolution biomolecular structures using cryo-EM [2,3], and the discovery of the structure of the ribosome [4,5], to name just 3 examples.

Research institutes occupy a specific niche in the larger research ecosystem. Their success can be measured by scientific contributions in the form of novel ideas, publication output, and grant funding. Furthermore, success is assessed not by shareholders but primarily by other scientists, both in a broad community sense and in terms of advisory boards, funding bodies, and so on. Other measures of success include the satisfaction levels of staff and trainees, their career development to go on to contribute to society in research or other venues, and commercial impact, for example. Selected examples of research institutes, their histories and characteristics can be found in Box 1.

**Box 1. Research institutes and their characteristics**

The success of the research institute model is exemplified by biomedical research institutes. One prominent example is the Laboratory of Molecular Biology (LMB) in Cambridge, United Kingdom, which was established by the Medical Research Council in 1947 and was the PhD training ground and dozen-year long workplace for one of us (SAT). The LMB boasts 12 Nobel Prizes and revolutionary breakthroughs such as key contributions to the discovery of the DNA double helix structure. Similarly famous is the European Molecular Biology Laboratory (EMBL), whose successful PhD program was the training ground for another one of us (FB) and boasts 3 Nobel Prizes. This intergovernmental research organization was founded 1974 and provides amazing opportunities to group leaders to perform ground-breaking research through generous core funding in a completely free, blue skies research environment. As an example of a more recently established institute, the Howard Hughes Medical Institute (HHMI) Janelia Research Campus in Virginia, United States of America was opened in 2006 and has an interdisciplinary approach ranging from mechanistic cognitive neuroscience through to the new “4D cell physiology” program. Its philosophy is to create a culture of collaboration with freedom to pursue research.

Many institutes have a dominant scientific and/or technological culture, be it structural biology (the LMB), genomics (the Wellcome Sanger Institute in Hinxton, UK), or neuroscience (HHMI Janelia Research Campus or the German Center for Neurodegenerative Diseases (DZNE) in Bonn, Germany). In addition, a “founding myth” and research culture, often related to the original leadership, is often woven into the institutes’ identity. For example, John Sulston propagated an open science and open data spirit at the Wellcome Sanger Institute, with a strong emphasis on team science, whereas Paul Nurse has built the Francis Crick Institute in London, UK into an environment supporting both basic and translational research and fostering the collaboration across both areas. The LMB identity is often associated with decades-long dedication to a single large scientific challenge, exemplified by Max Perutz’s solution of the structure of hemoglobin; additionally, Perutz’s manner of interacting with LMB colleagues in an informal, open, and nonhierarchical way feeds through to the institute’s culture today.

An important question that has grown in importance in recent years is how research institutes can promote a positive research culture. To us, research culture includes how staff and trainees interact with each other across the institute, what the institute defines as valuable and important, and what its definitions of success are (e.g., discoveries, papers, grants, spin-offs, training the next generation). It is influenced in both top-down and bottom-up ways: Leadership can set the tone and the values of the institute, while younger researchers, if well-integrated and listened to, can influence the institute’s direction and give the institute energy and excitement. It is also not just about people: Positive research culture can be incubated by the physical structure of the institute, in venues for social eating, sports, and after-work socializing, for example, as well as by regular events such as retreats to promote team building and coherence. The bottom line is that research culture is not just an added benefit for an institute: it can either nurture or impede the creativity of individual scientists, and as this creativity drives scientific discoveries, institutions are increasingly taking it seriously (see [6,7] for insightful discussion).

Thus a key question is how to incubate an inspirational research culture where productivity, ambition, and high-quality science are encouraged in a balanced, supportive, and inclusive way. While scientific output is generally perceived as the result of work by bench research scientists alone, there are multiple structures within a research institute involved in research delivery, and thus everyone in an institute is part of the research mission and should be recognized for their contributions. What are the ingredients required to craft a successful, collaborative, supportive, and thriving research environment? Below, we outline some key interacting components, which are summarized in Table 1.

**Table 1:** Key ingredients for a successful research institute.

Organizational component	Key ingredients
Funding review process	• Individual principal investigator-based review or collective (e.g., departmental) reviews depending on the institute • Review process designed to promote collaboration and interdisciplinarity • A carefully appointed scientific advisory board • A transparent and clear process
Administration and corporate services	• Proactive service mindset, customer-friendly • Fast turnaround and agility • Transparency in performance • Buy in to the institute’s vision • Clear two-way communication channels
Core facilities	• Proactive and transparent user data distribution • Rapid training • Equipment shared across the institute • Fair governance • Commitment to career pathways, acknowledgement in papers
Technology transfer and innovation teams	• Management of intellectual property, external partnerships, and patents • Legal and business counsel • Promotion of entrepreneurship in researchers
Training	• Train scientists at all levels across the institute • Research methods and cutting edge techniques • Holistic skills: communication, research management, leadership, etc.
Faculty recruitment	• Transparency • Internal or external recruitment processes (and measures that counterbalance any potential negative consequences) • Attractive packages • Long-term versus short-term considerations • Distribution of experience
Institute culture	• Transparency in operations and management/governance, recruitment, packages, salary, space, and platform access • Promote collaboration and creativity by fostering an open research culture (e.g., open seminars with unpublished work, retreats, funding mechanisms that promote collaboration) • Provide training at all levels: scientific as well as communication and leadership • Enable people to call out bad behavior (e.g., bullying, scientific fraud), with clear escalation routes and consequences, even for highest levels of management • Culture of allowing mistakes • Build diversity in the workforce at all levels based on merit • Supporting needs of diverse groups (parents, religious groups, individuals with disabilities, etc.) in order to build truly inclusive environment • Senior leadership buy-in to demonstrate commitment from the top

## Considerations for a successful research institute

**Fig 1:**
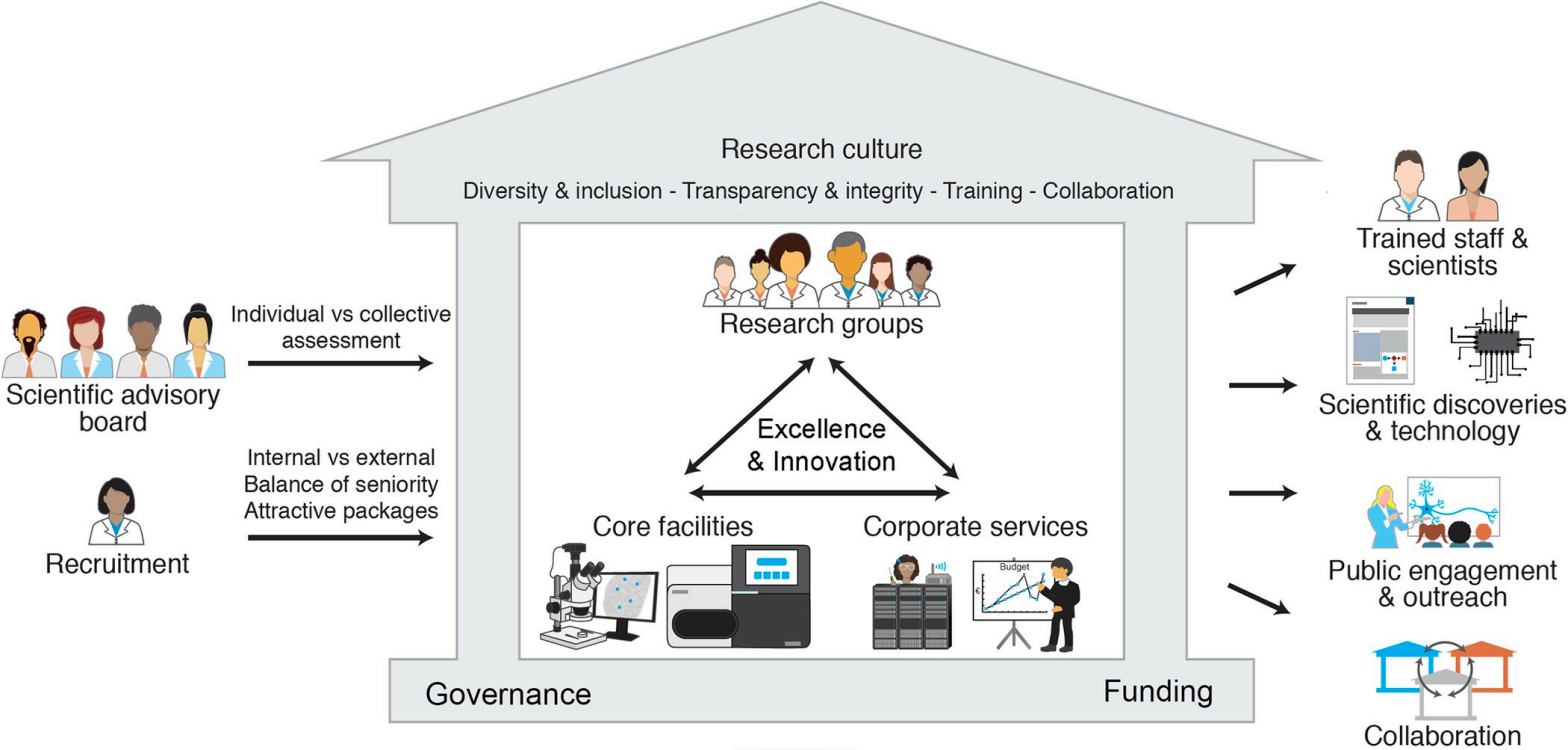
Ingredients for a successful research institute. Institutes are built on a framework of funding and governance indicated in the floor of the building, which can be varied depending on the institutional model. To build and maintain the institute, strategic recruitment is important, as are supportive and constructive advisory committees (shown on the left-hand side). For a research culture that fosters productive and creative science, continuous open communication and collaboration is key between the 3 basic components within the institute: faculty research groups, facilities and platforms, and administration and corporate services. Varied types of outputs of a successful research environment are shown on the right-hand side. (Illustration credit: Christina Usher.).

While there is not a single recipe for an ideal institute, every aspect of the “village” can influence the progression of the next generation of scientists and the discoveries they produce. Core facilities, a supportive administration and research groups form the golden triangle of a well-run institute (Fig 1). Within the golden triangle, research institutes need to consider the following concepts to maximize their success (explored in detail in the following sections):

- Listen, inside and out. Institutes need feedback mechanisms, both internal and external, to continuously evolve and optimize their organization and science.
- Enable scientists to focus on the science. Institutes benefit from effective, proactive, and communicative underpinning administration with a deep understanding of research culture, to free up scientists to focus on their science.
- Promote “plug and play” research. Effective and agile facilities and operational infrastructure, with state-of-the-art of the equipment, can be tremendously enabling for research.
- Build a holistic research environment. The institute needs a supportive research culture that empowers scientists to develop and realize their potential and promotes creativity.

While institutes are run in different ways and have different purposes, consideration of these key ingredients will help them to thrive.

### Listen, inside and outside: Effective feedback mechanisms

Effective feedback mechanisms help research institutes assess how well their progress is aligning with their goals. Perhaps the most important source of feedback to the leadership is from staff working within the institute, who will have direct knowledge about how well the institute is running on a day-to-day level. Open channels of communication for staff are thus vital: this could be to their direct line managers or as regular (and optionally anonymous) surveys. These can then feedback into internal assessments on a more formal level; for example, annual reviews can showcase not only scientific achievements but also policies regarding staff wellbeing that have been enacted following feedback.

One of the key external sources of feedback comes from funding reviews and assessments, which can focus more on the achievements of individual research groups (such as at the LMB or at the Max-Planck Society institutes in Germany) or on entire departments or programs (such as at the Wellcome Sanger Institute or at the Helmholtz Association institutes in Germany). The kind of assessment regime chosen may influence the science that happens in the institute. More individual-based reviews have the benefit that responsibility lies very clearly with an individual principal investigator (PI), but intra-institute collaborations are not specifically incentivized. Of course, individual groups can independently reach out to potential collaborators, but the institute leadership needs to closely monitor how such interactions and scientific collaborations develop. The leadership might also influence collaboration by implementing a PhD and postdoc program for training in the latest techniques in the different research areas or provide a venue for cross-pollination of different ideas to stimulate intellectual creativity and innovation. Crossing disciplines does come with risks as well as opportunities [8], especially if the groups do not speak a common language, and again leadership can step in in these situations, providing opportunities as well as monitoring the effectiveness of interactions (occasional joint review meetings with members of leaderships of across disciplines may help to facilitate this).

By contrast, the more collective-based review of entire departments or programs may incentivize intra-institute collaboration. This facilitates and expedites delivery of large research programs with a single common goal, as epitomized by the concerted contribution from the Sanger Institute to the Human Genome Project [9] and the Human Cell Atlas [10], and the Rhineland-Study at the DZNE [11]. To make sure that individuals from different fields will be motivated to interact with each other around the shared project in such collective programs, leadership needs to provide opportunities for interactions and ways of monitoring their effectiveness. Large projects with relatively well-articulated goals can catalyze development of technologies and provide data sets that individual laboratories can build on in the future (advances in genome sequencing during the Human Genome Project is a clear example of this).

Regardless of whether a research institute adopts individual or group evaluation, it is crucial to have a transparent and clear process that is communicated upfront to all parties within the institute and to the evaluation committee. The process should be proportional and efficient, as limited in time as possible, and occur with as little possible disruption to the research at the institute. In addition, a separate, carefully appointed standing scientific advisory board that engages in constructive feedback can provide vital and complementary comments. Such advisory boards ideally contain 1 or 2 members with long-term knowledge of the institute’s history and culture, who truly know the organization. Their sustained input can be vital, especially for new institutes still finding their feet. Finally, it is worth considering widening the net in terms of guidance and feedback to include wider society (for an example, see Box 2). We see engagement with wider society as a two-way dialog, with both parties benefitting [12].

**Box 2. Examples of successful practices by research institutes**

**Expand feedback**

- The Centre for Genomic Regulation in Barcelona, Spain engages citizens and stakeholders to guide and co-create its long-term research strategy_._

**Support core facilities**

- EMBL’s ARISE program of fellowships supports training of highly educated research infrastructure scientists. Researchers who want to stay in science but not on the tenure track are provided with an opportunity to learn the skills necessary to develop and maintain infrastructure, and core facilities will benefit from hiring well-trained staff.
- Cross-Europe initiative Core for Life brings together core facilities managers to together define best practices, provide training, validate and share technologies, and advocate for the importance of funding for core facilities.

**Boost technology transfer**

- Yeda commercializes the intellectual property generated by scientists at the Weizmann Institute in Rehovot, Israel.
- The Vlaams Instituut voor Biotechnologie (VIB) Innovation & Business team (Ghent, Belgium) bridges the institute’s science and industry by establishing patents, intellectual property licenses, industry collaborations, and spin-off companies.
- The Tri-Institutional Therapeutics Discovery Institute in New York, USA helps researchers from 3 biomedical centers translate their discoveries to preclinical studies.

**Expand trainee experience**

- Many organizations, such as EMBL, provide PhD training programs across departments/sites, increasing exposure to multiple disciplines.

**Improve equity, diversity, and inclusion**

- The Instituto Gulbenkian de Ciência in Lisbon, Portugal runs the António Coutinho Science Awards, which provide fellowships and research funding to citizens of Portuguese Speaking African Countries (PALOP) or descendants of those from PALOP countries.
- The Wellcome Sanger Institute recently launched the three-year postdoctoral Excellence Fellowships, which are aimed at people from Black heritage backgrounds.
- The Athena Swan charter can help institutions achieve gender equality objectives and provide structures for self-assessment.
- The Wellcome Sanger Institute has a Behavioral Competency Framework (BCF) that all employees sign up to and which defines the behaviors expected and encouraged in the entire institute.
- International graduate programs such as Taiwanese International Graduate Program foster diversity by recruiting PhD students from across the world.

**Network across institutes**

- EU-LIFE is an alliance of leading life sciences research centers from 15 European countries, which forms a strong advocacy voice to influence policy, share best practices, and inspire open and ethical science.

### Let scientists focus on the science with a proactive and communicative administration

While all scientists love discussing their new discoveries and next experiments, and some also like to spend time developing longer term scientific strategies, very few are passionate about administrative organizational strategy. This may be a mistake given the importance of an efficient administration for the smooth running and success of a research institute [13]. While institutes rightly seek excellence in faculty recruitment, they should also invest effort into recruiting and training excellent administrators.

The administrative apparatus needs to balance the desire of scientific leaders for unconstrained freedom with the necessity of managing budgets and applying business principles and governance in often publicly funded, not-for-profit organizations with liabilities and legal obligations. Finding the balance between these 2 objectives is challenging. For administration to operate harmoniously with research groups, dedication to the research culture of the institute and a proactive approach to enable science are foundational. Administrative staff need to understand that speed and flexibility can make all the difference in science. Every employee joining the administration should be made aware of the scientific research process in the institute. What are the results, discoveries, and outputs of scientific research? Why does timeliness matter? Administrative staff should understand the importance of rapid and nimble responses when providing scientists with working instruments, tailored contracts, and efficient and understandable guidelines. Supportive administrative staff will proactively provide information and co-develop (with researchers) streamlined processes and standard operating procedures to help the scientists achieve their research goals while complying with rules and regulations.

A bottom-up, proactive administration culture can be usefully complemented by a top-down organizational strategy. The administrative leadership needs to clearly articulate the vision and overarching goal of rapid and nimble research support, empowering proactive and creative problem solving by all administrative staff members. A key element of this is the level of tolerance with respects to mistakes. Getting the balance right between agility, speed, and risk aversion is important for administrative teams.

To help achieve this mindset and culture, a “liaison” system between administrative team heads and faculty members can be remarkably effective. Each administrative department can have research group leaders who act as contact points, resulting in a bidirectional dialog and exchange of information. Similarly, when faculty members understand the mechanisms and constraints of administration, they can recognize when and how to request changes effectively—communication is vital.

To develop a proactive administration who are aligned with the research aims and culture of the institute, administrative staff must feel welcomed and valued. However, administrators commonly feel their skills are underappreciated [14]. Thus, it is key to communicate with respect and to publicly acknowledge and support the vital work that administrators do.

It is impossible to overemphasize the importance of communication to an institute. Internal communication has a key role in its day-to-day functioning: top-down, it allows all staff to stay informed of the long-term goals and strategies of the institute; bottom-up, it allows leadership to stay abreast of the research advances and practical concerns of the workforce; and sideways, across the institute, researchers greatly benefit from knowing what their neighbors are doing (this can help foster collaborations). Smooth and positive communication across roles enables a committed and effective research culture to flourish.

External communications, on the other hand, ensure that discoveries are not confined to the pages of scientific journals. As many institutes are publicly funded, they have a duty to convey their discoveries to the public in relatable and exciting ways. Thus, a well-supported communications office can be a great help to any institute. Communication professionals can act as the bridge between scientists and the media, help write press releases and create audiovisual output, and train scientists in how to tell their research stories. External communication also has a key role in defining an institute’s success and promise to potential funders. In certain regions, philanthropic donations can make up a significant portion of an institute’s income, and therefore, many institutes have dedicated philanthropy departments to manage and promote this activity. Here, a key aspect is donor engagement: the institute needs to communicate the impact of donations, which may be in the form of events, lay reports, or lab tours, for example.

### Facilitate “plug and play” research with facilities and operational infrastructure

Core facilities, comprising technical instrumentation, methods, and the expertise of highly trained staff, enable faculty teams to focus on new areas of scientific development while benefitting from services such as next-generation sequencing, microscopy, bioinformatics, and research software engineering. Core facility teams often implement cutting-edge methodologies for the benefit of the whole institute [15], and shared core facilities can offer efficiencies and economies of scale. Indeed, core facilities can act as “force multipliers” for various essential aspects of the institute, from recruitment and retention to grants and knowledge production [16].

To develop the broad expertise needed to optimally exploit new technologies, community and collaboration are key. For example, faculty teams and core facility staff can work closely together to develop, optimize, and scale-up a new technique or methodology. Just as we discussed with regards to administration, it is crucial to get core facility staff on board with the vision of the institution and to publicly acknowledge their work, such as via authorship and/or acknowledgements in papers and talks, and in events or awards. Two examples of endeavors to support core facility staff are outlined in Box 2.

The governance of core facilities is an important consideration: researchers must be ensured fair access, and a balance must be struck between adopting cutting-edge techniques and maintaining a high level of robustness and reliability in results and data delivery. A steering committee structure can maintain oversight of the facility, and a transparent online system can monitor services provided and usage statistics, turnaround times, and key performance indicators. Depending on the research focus of the institute, some facilities can grow to be exceptionally large and need a more defined structure and management. For example, research institutes that deal with large genomic data packages and huge sample sizes require specific adopted management schemes (for an informative case study, see [17]).

### Build a holistic environment to empower individuals to develop and realize their potential

Research institutes are not merely spaces where science is done. Rather, they can proactively accelerate research advances and enhance the day-to-day lives and career prospects of the researchers within. This can be in more structural/formal respects (such as recruitment, technology transfer, and training) and also in social/cultural dimensions that generate a positive environment where ideas and creativity thrive.

### Recruitment

Research is fundamentally about the people involved, so recruiting talented staff that contribute to the institute aims and culture is vital. Hiring decisions have far-reaching implications for the institute in terms of both science and culture, and this applies across the board for anyone hired in any capacity. Institutes deal differently with recruitment, ranging from an internally driven process (e.g., Wellcome Sanger Institute) to a largely external assessment (e.g., EMBL, where hiring committee members are drawn from across the international European Molecular Biology Organization membership). Each have their benefits and risks.

In internally driven recruitment, the institute and its leadership are in complete control of individual promotions, recruitment, science, and personnel strategy. The vision and strategy of the leadership team is executed in a top-down manner, with the advantage that decisions can be made quickly. At the same time, unchallenged views on strategy and personnel selection may allow bias and nepotism to creep into the process. To counterbalance this, human resources departments have a key role in ensuring well-managed recruitment campaigns, and the external scientific review process provides further checks and balances. External assessment has the advantage of objectivity and potentially avoiding personal issues and local politics. Recruitment by hiring committees with eminent external members may help increase trust in a system. Potential disadvantages include the risk of “parachuting” someone in who does not understand the research areas and organizational context of the current faculty and also a risk of consensus decisions that promote orthodoxy in hiring. There may not be a single ideal way of conducting recruitment in a given research institute—what is key is an awareness of the pitfalls of both approaches and taking steps to counterbalance them.

Once hired, scientists rightly want to be fairly renumerated for their efforts. While an attractive package is a nonnegotiable factor in decisions about where to work, extra perks such as on-campus childcare or residential accommodation can make a huge difference to the lives of scientists. Differences in pay within the institute need to be well justified based on experience and delivery. Maintaining fairness and transparency is crucial in order to avoid pay and resource gaps that are not justified based on scientific productivity, such as the gender pay gap (for a recent survey of this gap in the UK, see [18]). Finally, the option of dual hires (where spouses are hired to the same institution) can be a huge incentive for a candidate to accept an offer, particularly if they are coming from a different country.

A key question is whether to hire new PIs on tenure-track or no-tenure models. The dynamically changing workforce that no-tenure models can generate might avoid intellectual stagnation and help the leadership adapt to new challenges with new hiring. At the same time, new PIs may be put off by the lack of long-term security and be more attracted to tenure-track models. To counteract this, research institutes can extend time periods of fixed-term contracts and offer more generous core funding. In our experience, a good distribution of experience contributes to a stimulating research environment: junior group leaders bring energy, dynamism, and a fresh, sometimes revolutionary perspective, while senior group leaders contribute mentorship, experience, stability, and strategic perspectives. A healthy balance of faculty members with different levels of experience, without a concentration of only junior or only senior group leaders, may thus be a common aim.

The number of faculty is also a key consideration. There is no “ideal” institute size because more or less staff may be required depending on the research questions and funding environment. While smaller institutes may appear to provide a more convivial atmosphere, the social “tone” of an institute of any size can be influenced by leadership style and work culture, as discussed below. Related to institute size is group size. While some institutes restrict group size (e.g., the Francis Crick Institute and the LMB), others have no explicit limit. Small groups can have effective communication and teamwork, and their leanness can give a greater focus on a particular problem, whereas large groups are able to be interdisciplinary, with critical mass in 2 or more areas, and provide the benefits of research done at scale. Indeed, research as a whole flourishes with a diverse range of group sizes, with large groups expanding and building up research, and small groups starting new areas of inquiry [19].

Whatever the size, institutes work best when grouped around either a technology (e.g., the Structural Studies Division at the LMB) or around a biological topic (e.g., neurodegeneration at the DZNE). This leads to the question of how to manage the extent of interdisciplinarity within an institute. Focus on a single discipline might inhibit creativity, while too much interdisciplinarity might lead to a lack of overlap between research groups, with no benefit from synergies. The best space might therefore be somewhere in between [6].

Individuals are what make any institute run—science is really about people and their day-to-day interactions. This means that recruitment can make a huge difference to the “feel” of an institute. When hiring new faculty members, scientific capability is of course key, but not the only aspect. How well will this personality merge with the existing faculty? Alternatively, are they too similar, so would the institute benefit from disruption of the status quo? Do they fully buy in to the research culture and vision that the institute is striving for? Individuals with energy and drive can further energize others, provide inspiration for students and postdocs and, in turn, make the institute an attractive place to work for future hires. Identifying such individuals can thus be key, particularly in the early stages of an institute’s life.

The appointment of a new director is one of the most influential recruitment decisions, as they act as a figurehead for the institute, externally and internally. The new appointee might take the institute in an entirely new research direction or aim to develop a new kind of research culture: the appointment itself is a statement of the future vision of the institute. What counts as an ideal candidate will of course depend on an institute’s priorities. Is scientific excellence paramount or administrative experience? As discussed above, the decision between hiring from inside or outside will also have great implications. While an insider may understand the institute’s unwritten rules and be able to quickly identify key issues, an outsider may bring a fresher view, with novel ideas and management qualities. Personal style is crucial here, but overall leadership (of any organization) is fundamentally about serving the mission and the people in the institute. Directors who are exceptional in this regard have often been founders or early directors of institutes (e.g., Janet Thornton, an early director of the EMBL–European Bioinformatics Institute), perhaps because they feel so closely intertwined with the institutes’ identity and people.

### Technology transfer and training

Once staff are in place, there are various ways that research institutes can build a supportive and inspirational research environment. As hubs of innovation, institutes generate novel ideas and inventions, and to ensure that these findings are protected and taken forwards for the benefit of society, a technology transfer system can manage intellectual property and the process of licensing and commercialization. Innovation and business development teams can also educate researchers to promote an entrepreneurial attitude, and institutes in turn must make space for, recognize, and reward activities such as the founding of start-up companies. This could be via reward and incentives through sharing of royalties from patents or the use of employment contracts with a percentage of time given over to entrepreneurial activity. Examples of successful technology transfer are shown in Box 2.

In addition to helping scientists achieve impactful discoveries, forward-looking institutes will train scientists at all levels in the key skills required for the scientific workplace, but also in communication, research management, and leadership. Scientific training will allow researchers to stay up to date with cutting-edge methodologies. To prepare students and postdocs for life as an independent investigator (if this is the path they choose), research institutes can provide access to training in writing papers and grants, project and lab management, and science communication/public engagement. PhD programs that provide training across departments and sites can be extremely useful and stimulating, exposing students to various techniques and scientific perspectives early on in their training that are often exploited in subsequent thesis work and later careers. An institute’s success may be measured in large part by the impact of its trainees on society, and a well-supported training and outreach program can be invaluable to this.

The mentorship of younger scientists by more senior staff in the institute can be crucial to their career success. As well as providing career guidance, compassionate mentors outside of the host lab can help to tackle research misconduct in its many forms. Young researchers should have trust in the wider system and know that they can raise concerns without having to challenge their supervisor directly: a supportive mentoring network can provide this.

### Building an inclusive environment

One of the beauties of working in science is encountering people from different cultural backgrounds, and research institutes should aim to integrate different personalities and characteristics, fostering diversity, and viewing it as a strength. An inclusive environment brings out the best in scientists and science [20,21], and different ways of thinking contribute to greater creativity. Thus, diversity in scientific approaches is more likely to be found among a group of diverse individuals, and a successful research culture takes equality, diversity, and inclusion seriously. As we have argued with respect to gender equality [22], this must be built in at the policy level so that the system is organized to incentivize good behavior. We are also encouraged by the efforts of funders in this regard, such as Wellcome [23] and HHMI [24]. Going forwards, research institutes need to further develop and promote inclusive policies encompassing areas such as equality of opportunity, parental leave policies, protection from bullying and harassment, and sustainability. There should be public, high-level buy-in for these policies and dedicated funding streams.

There have been numerous recent examples of research institutes implementing policies to promote equality, diversity, and inclusion in locally relevant ways (Box 2). Ensuring diversity on management and advisory boards is another aspect that needs to be developed to achieve a greater diversity in the workforce at all levels in the long term.

The majority of discussions about diversity of science (inevitably including those covered in this piece) are centered around helping institutions in Western nations increase diversity. The risk is that this is less relevant for other institutes, particularly those in nations where English is not commonly spoken. For such institutes, a bigger problem may be to attract top science talent from the global marketplace. A highly funded institute in a wealthy nation may appear “more diverse” because it has the luxury of attracting global talent, and the definition of what constitutes “diverse” will differ from country to country. While we do not explore these issues further here, we hope this article spurs responses from colleagues around the world, who might share their local experiences for research institute success. Discussions with colleagues based in Taiwan show how institutes there have established international PhD programs (Box 2) and targeted recruitment of faculty and guest professorships to access the global talent pool.

Wherever the institute, a positive research culture can be helped by a clearly articulated code of conduct (Box 2). Such frameworks should be developed with the staff of the institute, should be open to change over time, and be flexible enough to accommodate the various cultural backgrounds and personalities that necessarily make up a modern research institute. Rather than being a restrictive straight jacket or homogenizing tool, they should be a way of recognizing and fostering behaviors that contribute to a positive research culture without suppressing personal authenticity and freedom of speech. Indeed, there are many different types of people that can be accommodated and contribute to a team. Exciting research often occurs on the fringes: There is a risk that research institutes stifle those with unorthodox views who may not appear to conform to standard expectations of behavior or research direction, yet bring unique strengths and perspectives to the table. The challenge for leadership is how to accommodate researchers with potentially difficult characteristics within a broadly inclusive and tolerant environment.

Finally, a positive research culture must have staff wellbeing at the core: just as an institute must ensure staff safety via a dedicated health and safety program, staff mental health should also be high on the radar of institute leadership. There are various ways in which mental health can be supported (for a recent perspective, see [25]).

## Meeting today’s and tomorrow’s challenges

Research institutes today have to meet a host of new, often interlinked, challenges that stand in the way of their ability to transform society with scientific innovation. A first challenge is simply to get the necessary funding to carry out research. Many institutes have found their funding curtailed in recent years (e.g., due to austerity programs that reduce government spending on research or inflationary pressures). To help counter this, prominent institutes can act as vocal advocates for science funding. A well-run communications department and publicly vocal and compelling director will undoubtedly help in this regard. Institutes can and should be in the public conversation, helping to counter disinformation and increase public trust in science, which ideally will feed back to politicians allocating budgets. Elsewhere in the world, research funding has been a persistent issue that will likely continue into the future. While philanthropy or global funding sources are potential solutions, another option is to expand international collaborations between institutes (taking inspiration from EU-LIFE, see Box 2). In this way, people, skills, and knowledge would move between countries in a way that strengthens local capacities. Is the time ripe for a United Nations of Research Institutes?

The COVID-19 pandemic was a global challenge that changed the way all research institutes worked. While the short-term effects were seismic—institutes shut for months at a time during lockdowns, freezing whole programs of research—there were also more lasting impacts, notably the naturalization of hybrid or remote working. This brings its own challenge: how can we foster team cohesion when interactions are mainly virtual? Online conferences are often less interactive than in-person ones, leading to fewer of the chance meetings that seed many of the innovations of the future (for discussion, see [26]). The risk is that the same might occur within research institutes, if the corridors and cafeterias are not filled with the sounds of scientists discussing their latest data. On the other hand, the virtualization of science can increase inclusivity, both at local and global levels (e.g., those with caring duties can work from home, while cutting-edge science can be shared virtually worldwide to expand knowledge transfer). Overall, for an institute, maintaining a strong identity and research culture may always rely on people meeting in person, but harnessing new virtual technologies to increase remote interactions (e.g., in virtual reality) can also complement this. A great challenge in the future therefore is how to maintain the physical–virtual balance.

A global challenge that will only increase in coming years is the climate crisis. Many scientists are increasingly uncomfortable with flying for work (for discussion, see [27]). Institutes can also have a role here, for example, in mandating the number of international trips expected from faculty as part of wider carbon reduction or net zero policies. A slew of climate-related societal challenges will inevitably impact research institutes, including food shortages, changes in migration patterns, acute health impacts, and physical impacts on infrastructure such as flooding. The institute’s operational leadership will need to keep these impacts on the horizon to guide their choices. Just as important will be the psychological effects of a changing world on the people working within the institute: climate anxiety may, at least in part, be mitigated by strong and open climate policies at the institute level.

A final challenge—and opportunity—is the increasing inclusion of artificial intelligence (AI) into an institute’s work and culture. While it is still difficult to envision how AI will affect our approaches, we expect that we are at the beginning of a major change. For example, the newly developed ways to analyze data and literature using AI may provide transformative insights for research and research culture. We expect AI to change how we develop novel scientific ideas and concepts. Institute leadership needs to closely follow the challenges and opportunities that arise through this transformative technology, and importantly, involve all staff in conversations about how best to move forward.

Overall, a research institute has to balance a multitude of factors. It needs to support scientists to flourish and forge a career, while simultaneously adopting an overarching research strategy that relies on good team workers, efficient project/research managers, and good leaders of people. Holistic, collaborative, and responsive approaches are vital to create scientific villages that foster the next generation of scientists and continue to produce world-changing discoveries.
